# Detection of atherosclerotic plaques with HDL-like porphyrin nanoparticles using an intravascular dual-modality optical coherence tomography and fluorescence system

**DOI:** 10.1038/s41598-024-63132-6

**Published:** 2024-05-29

**Authors:** Rouyan Chen, Lauren Sandeman, Victoria Nankivell, Joanne T. M. Tan, Mohammad Rashidi, Peter J. Psaltis, Gang Zheng, Christina Bursill, Robert A. McLaughlin, Jiawen Li

**Affiliations:** 1 School of Electrical and Mechanical Engineering, Faculty of Sciences, Engineering and Technology, The University of Adelaide, Adelaide, SA 5005 Australia; 2 Vascular Research Centre, Lifelong Health Theme, South Australian Health and Medical Research Institute (SAHMRI), Adelaide, SA 5000 Australia; 3 Institute for Photonics and Advanced Sensing, The University of Adelaide, Adelaide, SA 5005 Australia; 4 Faculty of Health and Medical Sciences, The University of Adelaide, Adelaide, SA 5005 Australia; 5 Department of Cardiology, Central Adelaide Local Health Network, Adelaide, SA 5000 Australia; 6 Department of Medical Biophysics, University of Toronto, Toronto, ON M5G 1L7 Canada; 7 Princess Margaret Cancer Centre, University Health Network, ON M5G 1L7 Toronto, Canada

**Keywords:** Biophotonics, Nanoparticles, Cardiovascular diseases

## Abstract

Atherosclerosis is the build-up of fatty plaques within blood vessel walls, which can occlude the vessels and cause strokes or heart attacks. It gives rise to both structural and biomolecular changes in the vessel walls. Current single-modality imaging techniques each measure one of these two aspects but fail to provide insight into the combined changes. To address this, our team has developed a dual-modality imaging system which combines optical coherence tomography (OCT) and fluorescence imaging that is optimized for a porphyrin lipid nanoparticle that emits fluorescence and targets atherosclerotic plaques. Atherosclerosis-prone apolipoprotein (*Apo*)*e*^-/-^ mice were fed a high cholesterol diet to promote plaque development in descending thoracic aortas. Following infusion of porphyrin lipid nanoparticles in atherosclerotic mice, the fiber-optic probe was inserted into the aorta for imaging, and we were able to robustly detect a porphyrin lipid-specific fluorescence signal that was not present in saline-infused control mice. We observed that the nanoparticle fluorescence colocalized in areas of CD68^+^ macrophages. These results demonstrate that our system can detect the fluorescence from nanoparticles, providing complementary biological information to the structural information obtained from simultaneously acquired OCT.

## Introduction

Atherosclerosis is an inflammatory disease which is characterized by a build-up of fatty plaques in the arterial wall. Atherosclerotic plaques can undergo a rupture event that causes a thrombotic occlusion, resulting in sudden ischemic cardiovascular events, such as acute myocardial infarction or stroke^1^.

Critical disease processes in atherosclerotic plaque formation, such as infiltration of inflammatory cells, have been visualized with modalities such as optical coherence tomography (OCT), although with low specificity^2,3^. Visualization of macrophages has also been explored with OCT, however imaging can be confounded as the macrophages appear similar to several other relevant features, such as cholesterol crystals, microcalcifications and the vessel membrane^2,4^. Label-free approaches are currently under development that may provide insight into molecular information, including near infrared spectroscopy^5^, near infrared autofluorescence^6^, and time-resolved fluorescence^7^. An alternative approach is to augment structural imaging with an additional imaging modality that uses contrast agents, such as nanoparticles^8–10^, to visualize differences in the molecular composition of the plaque. These nanoparticles typically have ligands, such as antibodies attached to target specific receptors that are overexpressed in disease settings, hence enabling disease-targeted imaging.

Porphyrin-lipid high-density lipoprotein mimetic nanoparticles (Por-HDL-NPs) have been previously reported as a fluorescence contrast agent for guiding brain tumor resection in an in vivo animal model^11^. Por-HDL-NPs are composed of porphyrin-lipid and an apolipoprotein A-I (ApoA-I) mimetic peptide named reverse-4F (R4F)^11,12^. ApoA-I is the main protein component of high-density lipoproteins (HDL) that interacts with the scavenger receptor class B type 1 (SR-B1), which is highly expressed on macrophages in atherosclerotic plaques. The Por-HDL-NPs has shown to target SR-B1 in cancer settings, when compared to other nanoparticles, they show a long retention in the tumor, which was evident from the increased contrast in the tumor site over a 48 h time period after administration^13^. We have shown that Por-HDL-NPs track to atherosclerotic plaques through visualization of their emitted fluorescence in vitro in macrophages and in vivo in a murine model^14,15^. Furthermore, in the aforementioned study, the flow cytometry of the digested aorta of Apoe^**-/-**^ mice suggested that the largest population of porphyrin lipid positive cells were in the macrophage populations^15^. The in vivo fluorescence detection was performed using a small animal in vivo imaging system (IVIS), where fluorescence was observed in the plaque-containing aortic arch region^14,15^. However, using a larger, non-invasive imaging system such as the IVIS only provides a low-resolution view of the location of fluorescence, due both to the two-dimensional nature of IVIS imaging and the high optical scattering properties of fluorescence emission light in tissue.

Multimodal intravascular imaging provides an opportunity to achieve more localized, higher resolution imaging of pathogenic molecular processes in atherosclerotic plaque^16^. An example of this involves the use of highly miniaturized fiber-optic imaging probes that can be inserted inside the lumen of the artery, positioning the high-resolution imaging optics immediately adjacent to the plaque. This approach is currently used clinically with single-modality imaging such as OCT, where it allows visualization of anatomical features of plaques such as the fibrous cap and the necrotic core^17^. Intravascular imaging devices have separately been used with fluorescence imaging from non-specific contrast agents, such as indocyanine green^16,18^.

In this paper, we present a proof-of-concept study which demonstrates the use of a dual-modality OCT + fluorescence intravascular probe to characterize atherosclerotic plaques in mouse aorta. The probe is paired with Por-HDL-NPs, used as a contrast agent to characterize plaque molecular composition, and provide complementary information to the OCT signal. We establish that the highly miniaturized optics required for intravascular imaging can effectively detect the fluorescent signal from these nanoparticles and validate both nanoparticle-fluorescence and OCT structural measurements against a histological gold standard. Our findings demonstrate the feasibility of contrast-enhanced dual-modality intravascular imaging for plaque detection.

## Results

### In vitro imaging of macrophages treated with Por-HDL-NPs

**Figure 1:**
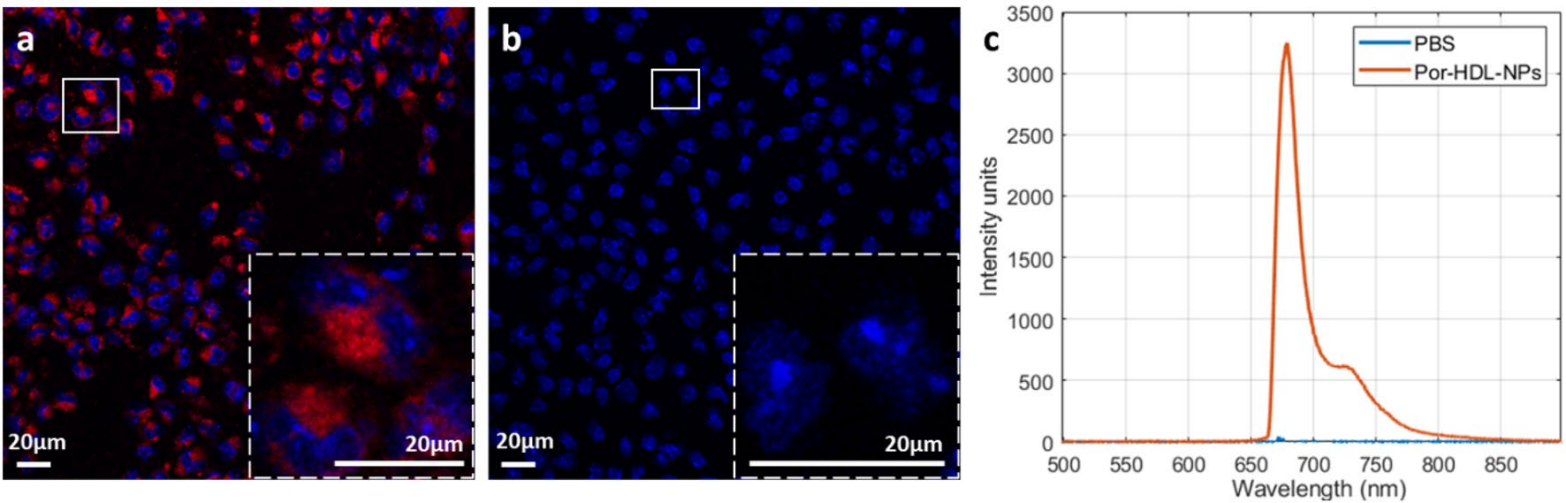
(**a**) DAPI stain of immortalized bone marrow derived macrophages (iBMDMs) incubated with Por-HDL-NPs, with an inset of zoom-in of the white box, blue: cell nuclei, red: Por-HDL-NP fluorescence; (**b**) DAPI stain of iBMDMs incubated with PBS, with an inset of zoom-in of the white box, blue: cell nuclei; (**c**) fluorescence measurement using a fiber-optic probe, orange: iBMDMs treated with Por-HDL-NPs, blue: iBMDMs treated with PBS.

To establish that the intravascular imaging probe and system can detect the nanoparticle fluorescence, we first conducted an in vitro macrophage cellular uptake study. Immortalized bone marrow derived macrophages (iBMDMs) were incubated with either Por-HDL-NPs or a vehicle control of phosphate buffered saline (PBS), then stained with 4′,6-diamidino-2-phenylindole (DAPI) nuclear stain and imaged under a confocal microscope. In iBMDMs incubated with Por-HDL-NPs, fluorescence was detected in the cytosol of macrophages using confocal fluorescence microscopy, demonstrating Por-HDL-NP uptake by the cells (Fig. 1a). In contrast, no fluorescence was observed in macrophages treated with PBS (Fig. 1b). Subsequently, images were acquired using our dual-modality intravascular fiber-optic probe. Fluorescence was detected in the Por-HDL-NP incubated cells, with its fluorescence spectrum shown by the orange line in Fig. 1c. No fluorescence was detected from the PBS incubated cells (blue line in Fig. 1c). The results from the intravascular imaging system validate the confocal microscopy results. These results demonstrated the ability of the dual-modality intravascular probe to detect fluorescence from Por-HDL-NPs in vitro.

### Intravascular imaging of atherosclerosis in the descending aorta of Apoe^-/-^ mice

We next examined whether the dual-modality OCT + fluorescence imaging system could be used for intravascular imaging of atherosclerotic plaques and Por-HDL-NPs in an atherosclerotic murine model. Atherosclerosis-prone *Apoe*^-/-^ mice were fed a high-cholesterol diet for 20 weeks to develop early-to mid-stage plaques. They were then injected systemically with Por-HDL-NPs 18 h prior to intravascular imaging.

**Figure 2:**
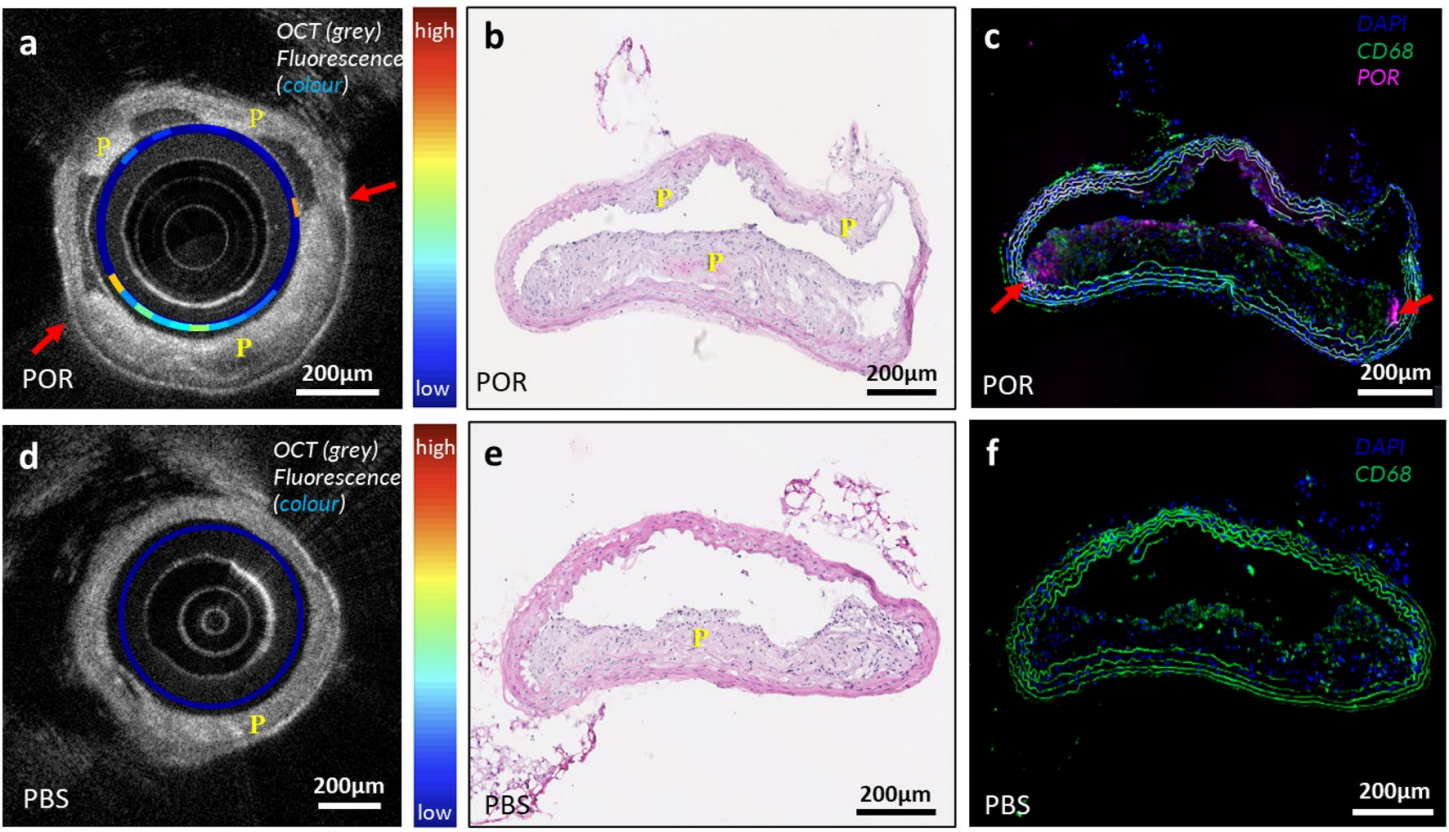
(**a**–**c**) Images of Por-HDL-NP injected mouse artery with plaques, (**a**) combined intravascular OCT and fluorescence images acquired with the fiber-optic probe; (**b**) corresponding Hematoxylin and Eosin (H & E) staining image, showing the general morphology of the plaque; (**c**) corresponding fluorescence microscopy image: DAPI stain of cell nuclei (blue), CD68^+^ macrophages (green), Por-HDL-NP fluorescence (violet); (**d**–**f**) images of PBS injected mouse artery with plaque; (**d**) combined intravascular OCT and fluorescence images acquired with the fiber-optic probe; (**e**) corresponding H & E staining; (**f**) corresponding fluorescence microscopy image: DAPI stain of cell nuclei (blue), CD68^+^ macrophages (green); P: Plaque.

Figure 2a represents an OCT image of a descending aorta from a mouse infused with Por-HDL-NPs. It shows the presence of plaque, labelled ‘P’, at three different sites in the OCT image, which are identified by intimal thickening. Furthermore, intravascular fluorescence detection is displayed as a colored ring superimposed on the OCT image, with the highest fluorescence indicated by the red arrows. After acquiring these in situ intravascular images, the descending aorta was excised to allow validation against a histological gold standard. When comparing the in situ OCT image with hematoxylin and eosin (H & E) stained sections (Fig. 2b), the locations of the plaques were well-matched. Using fluorescence microscopy (Fig. 2c) on the adjacent sections, it was found that higher fluorescence signals (in violet) were accumulated in the shoulder regions of the plaques, matching the location of fluorescence detected in the in situ images acquired with the dual-modality intravascular probe (indicated by red arrows on Fig. 2a, c). Lower fluorescence signals were also observed using intravascular imaging through the middle plaque situated in the 5–7 o’clock position of Fig. 2a, again matching the immunofluorescence microscopy image of the sections. The dual-modality intravascular probe was able to detect fluorescence signals across all the Por-HDL-NP treated mice throughout their arteries.

In PBS-treated control mice, no in situ fluorescence was detected by the dual-modality intravascular probe even at sites where plaque was present (Fig. 2d, e). This demonstrates that the in situ fluorescence measured by the dual-modality intravascular probe in those mice infused with Por-HDL-NPs, was specifically from the nanoparticle rather than the autofluorescence of the plaque. Similarly, no fluorescence was observed histologically in the aortas using fluorescence microscopy in the control mice (Fig. 2f).

**Figure 3:**
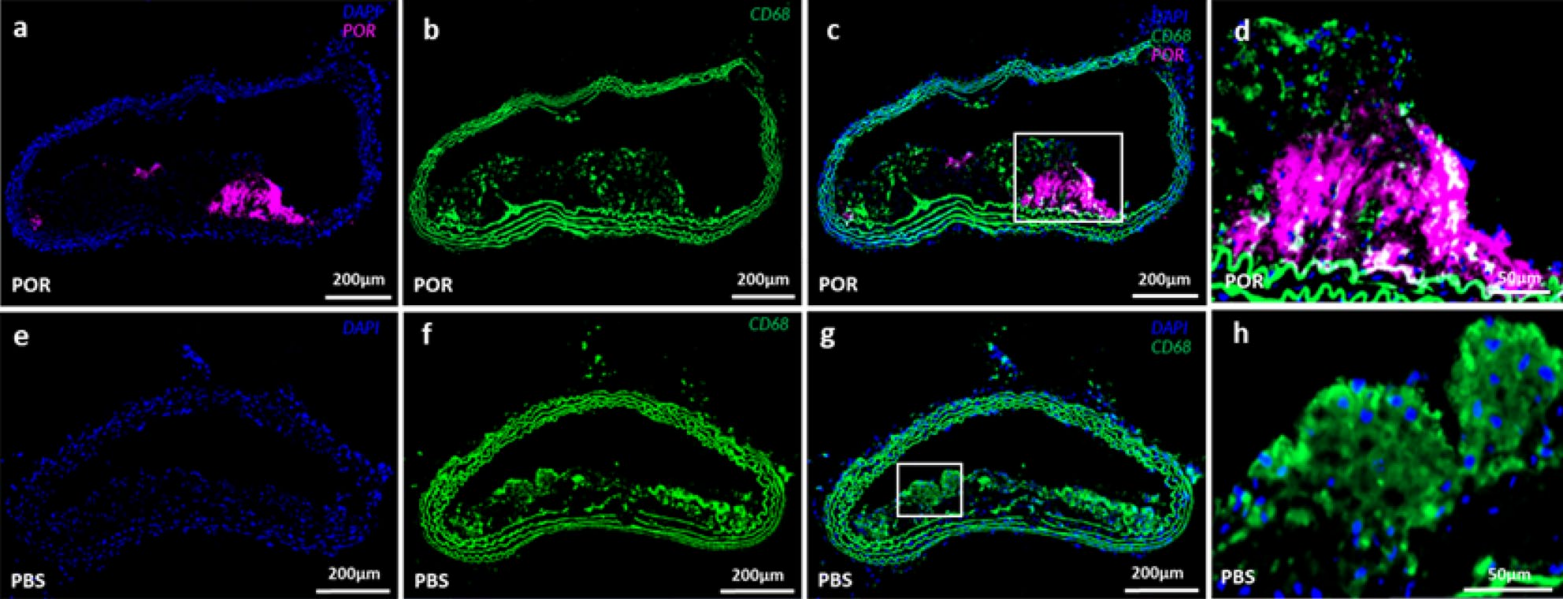
(**a**–**d**) Representative fluorescence microscopy images of Por-HDL-NP injected mouse artery with plaques, (**a**) DAPI (blue) and Por-HDL-NP (violet) fluorescence microscopy; (**b**) CD68^+^ immunofluorescence staining; (**c**) combined fluorescence microscopy image; (**d**) Zoom-in of white box in (**c**); (**e**–**h**) Representative fluorescence microscopy images of PBS injected mouse artery with plaque, (**e**) DAPI staining; (**f**) CD68^+^ immunofluorescence staining; (**g**) combined fluorescence microscopy image; (**h**) Zoom-in of white box in (**g**);

Immunofluorescence was used to identify CD68^+^ macrophages (green) within plaques (Fig. 3b, f). Por-HDL-NP fluorescence (Fig. 3a, c, d, in violet) was seen to colocalize with CD68^+^ macrophages (Fig. 3c, d in green). Colocalization was indicated by white color. This indicates that Por-HDL-NPs may have been taken up by the macrophages inside plaques, particularly those on the luminal facing side and in the shoulder region. It was also observed that not all macrophages colocalized with Por-HDL-NP fluorescence, suggesting not all macrophages internalized the nanoparticles. Although the elastic membranes of the artery appears to be positive (green) in CD68 staining, this is not caused by positive macrophage signals. Instead, it is related to autofluorescence of elastin which generates fluorescence signal under ultraviolet to green color light excitation (350–560 nm)^19^. The green color wavelength (475 nm) was used in the immunofluorescence imaging as the CD68 antibody was labelled with the Alexa Fluor 488 dye.

### Quantification of fluorescence measurement of Por-HDL-NPs

**Figure 4:**
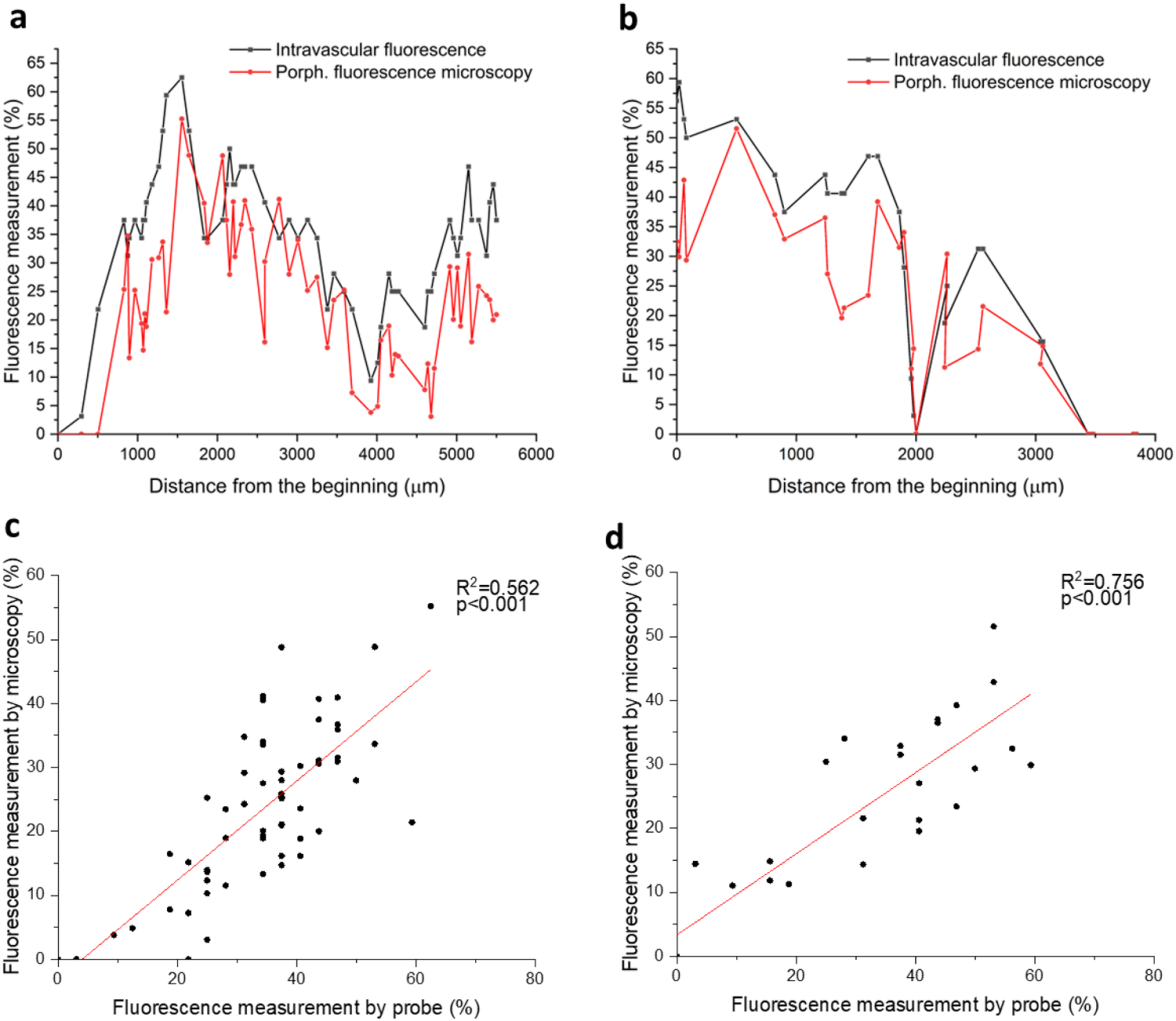
Comparison between the measurements of percentage of thoracic aorta plaque Por-HDL-NP fluorescence per frame by intravascular probe versus microscopy on co-registered thoracic aorta sections, for two independent Por-HDL-NP-infused mice. (**a**, **b**) Comparison plotted along the distance of the aorta; (**c**, **d**) comparison graphed in a scatter plot showing linear relationships.

We next quantitatively compared Por-HDL-NP fluorescence measured intravascularly against fluorescence present in co-registered, immunofluorescence histology sections. For each intravascular fluorescence image, 32 measurements were acquired over each full rotation of the probe. The probe was subsequently pulled-back to acquire these measurements at sequential positions along the length of the vessel. For each full rotation, we calculated the percentage of circumference of the lumen that showed 720 nm fluorescence emission above an empirically chosen threshold value. The same threshold value was used across all positions. In manually co-registered histological sections, we then calculated the equivalent measurement. To account for deformation of the vessel wall shape during fixation and processing, we calculated these measurements at regular intervals along the vessel wall, rather than at regular angles, as the cross-section of the vessel wall in the histological sections was generally no longer elliptical. Figure 4a and b plots the percentage of atherosclerotic plaque that was fluorescent, measured along the length of the vessels from two mice. We found corresponding increases and decreases in fluorescence measurements for both mice. Notably, the fluorescence measurements from intravascular imaging were generally higher for the corresponding measurements derived from confocal microscopy. We hypothesize that this is because of higher fluorescent signal emitted from the fresh in situ tissue, which might be negatively affected by tissue fixation and histological processing. Another possible cause of discrepancies in the fluorescence measurements can be ascribed to the limited number of axial scans (32 radial scans per image) that were acquired for individual intravascular fluorescence images. Such a coarse quantization can give rise to partial volume effects, where a small bright area of fluorescence gives rise to a positive measurement over a disproportionate large radial area. The low sampling rate of fluorescence can be attributed to limitations of the spectrometer used.

Analysis revealed a statistically significant linear correlation between signal from the dual-modality intravascular probe and fluorescence microscopy measurements for both mice (Mouse 1: R^2 ^= 0.562, *p *< 0.001 (Fig. 4c) and Mouse 2: R^2 ^= 0.756, *p *< 0.001 (Fig. 4d). We conclude that the dual-modality intravascular probe is able to assess the fluorescence emitted by Por-HDL-NPs in atherosclerotic plaques in situ.

### Quantification of plaque size measurement from OCT and H & E

We next quantitatively compared intravascular OCT measurements acquired with the dual-modality intravascular imaging probe against manually co-registered H & E histology sections. Whilst previous work has demonstrated the capability of OCT to measure the extent of atherosclerotic plaque^20^, this section establishes the accuracy of performing such measurements in a mouse model with a highly miniaturized probe (635 μm outer diameter). Similar to the fluorescence-histological validation, plaque size from intravascular OCT was measured as a percentage of the circumference of the lumen, and these measurements were taken at sequential locations along the length of the vessel. In terms of the corresponding H & E-stained sections, plaque size is reported in mm^2^ (plaque area), an accepted method of quantification in vascular biology^21^*.*

**Figure 5:**
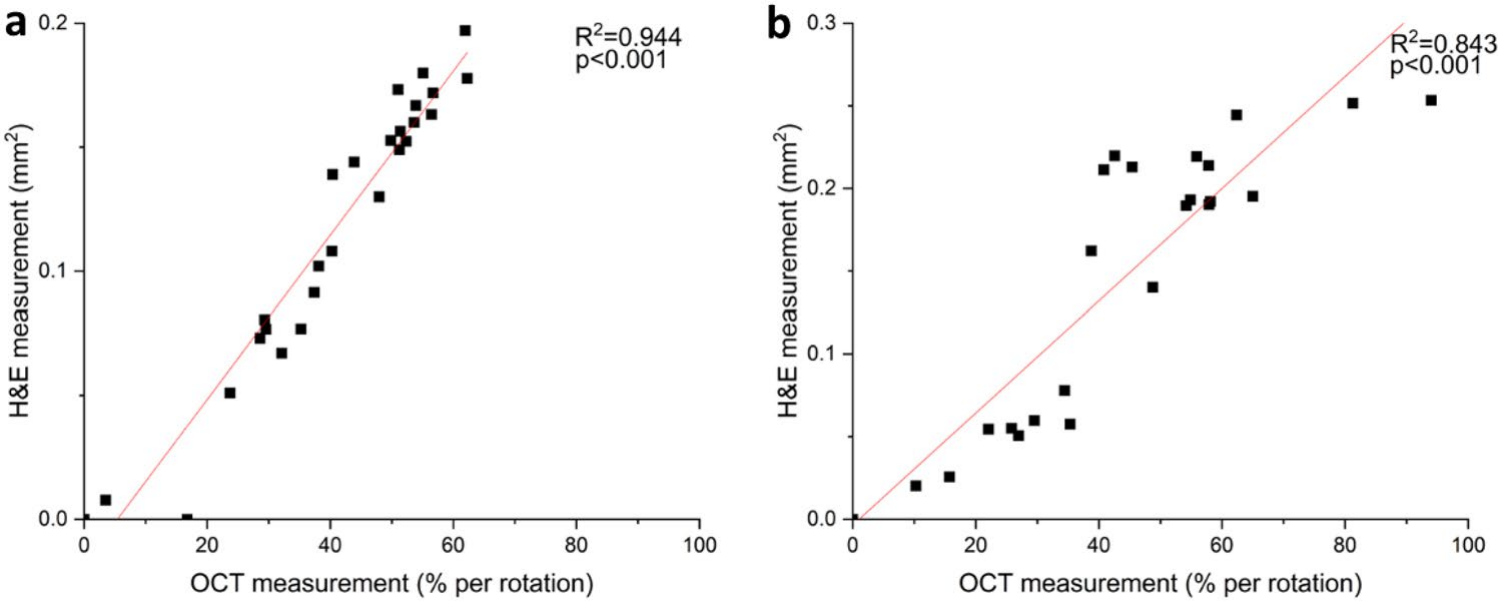
(**a**, **b**) Comparison of thoracic aorta plaque size measurements acquired by intravascular OCT probe versus aortic H & E-stained sections for two independent mice.

For assessment of the OCT images, the extent of the plaque was manually traced by a trained observer, blinded to the histological analysis, with the plaque identified by increased intimal thickening. In H & E histological sections, the extent of the plaque was identified by the thickening of the intimal structure as well as the presence of foam cells and a lipid core. The measurements of plaque area derived from OCT and histological analysis showed strong linear correlation (Fig. 5a, b), with R^2^ of values 0.944 and 0.843 for the two mice studied (both *p *< 0.001). Our findings suggest the OCT images acquired by our intravascular probe may be used to measure plaque area in a way that correlates with histological assessment.

## Discussion

Accurate detection of atherosclerotic plaques, especially their earlier stages of development, remains a significant unmet clinical need. Detection of early-stage plaque provides the best outcomes following interventions that reverse development and substantially decrease the risk of vascular complications such as myocardial infarction and stroke. This study examined the use of Por-HDL-NPs as a fluorescence contrast agent both in vitro in iBMDM cells and intravascularly within an *Apoe*^-/-^ atherosclerotic murine model. The in vitro study revealed the inherent fluorescence capability of the Por-HDL-NPs, which was detectable using our dual-modality intravascular system with a miniaturized fiber-optic probe. The acquired fluorescence spectrum was similar to that previously reported by Cui et al^11^, showing two peaks on the spectrum, one at 675 nm and the other at 720 nm. Plaque size and Por-HDL-NP fluorescence, detected intravascularly with the dual-modality intravascular probe, were able to be quantitatively analyzed and significantly correlated with co-registered measurements obtained histologically from thoracic aortic section images.

Various fluorescently labelled nanoparticles have been previously explored in animal models for detecting atherosclerotic plaques^8,22,23^. Often, nanoparticles administered in the blood stream are coated with polyethylene glycol (PEG) to facilitate prolonged circulation time^24^. Once infused, the nanoparticles enter the plaque through microvessels that extend into the plaque from the arterial adventitia or via permeable, dysfunctional endothelium that co-locates with developing plaques^25^. By virtue of their lipoprotein-like structure, which enables them to evade the immune system, Por-HDL-NPs have an extended circulation half-life that relinquishes the need for PEGylation. This extended half-life was made evident in the current study through our ability to detect Por-HDL-NP-specific fluorescence in atherosclerotic plaques 18 h post-administration by intravascular imaging and fluorescence microscopy. This represents a distinct advantage of Por-HDL-NPs over nanoparticles that require PEGylation, as PEGylation hinders cellular uptake and subsequent endosomal escape^26^, thus reducing the effectiveness of the nanoparticles, along with concerns about anti-PEG immune responses^27,28^.

Recently, there has been a focus on synthesizing nanoparticles that provide targeting to specific cell types that make significant contributions to atherosclerotic plaque development, including macrophages^29,30^ and endothelial cells^31,32^. Due to the incorporation of the R4F peptide that targets SR-B1, Por-HDL-NPs preferentially bind to macrophages, including atherosclerotic plaque macrophages that express high levels of SR-B1. Once bound, Por-HDL-NPs enter cells via receptor-mediated phagocytosis. This specificity of Por-HDL-NP uptake enables visualization of viable macrophages that are able to undergo receptor mediated phagocytosis in plaques, which can also be accurately quantified through fluorescence using our intravascular imaging probes. Furthermore, Por-HDL-NPs have a mechanism for preventing background fluorescence emission. Por-HDL-NPs have high density packing of porphyrin-lipid and therefore exhibit self-fluorescence quenching in their intact form whilst in the circulation^11^. Once internalized into cells and subjected to structural disruption, they then switch to an unquenched state allowing fluorescence emission from the porphyrin molecules. This feature allows for more accurate measurements in quantifying the viable macrophages in atherosclerotic plaques.

The first steps of atherosclerosis development involve the recruitment of monocytes to the endothelium that then migrate into the vessel wall where they differentiate into macrophages and take up lipid. Over time, excessive lipid uptake causes macrophages to become apoptotic and necrotic, at which point they are unlikely to be able to internalize nanoparticles; yet may still be detectable by CD68^+^ immunofluorescence. The fluorescence from these Por-HDL-NPs in plaque macrophages was captured by our intravascular imaging probe and by fluorescence microscopy, in parallel with CD68^+^ macrophage immunofluorescence histology. Examination of our fluorescence and immunofluorescence images of our thoracic aorta plaque sections revealed that Por-HDL-NPs are predominantly taken up by luminal facing macrophages, suggesting preferential uptake into the newly recruited macrophages that reside closer to the luminal side of plaques and are viable. This observation explains why the Por-HDL-NP fluorescence was reliably detected by the intravascular probe despite its limited focal length, as the nanoparticle fluorescence is localized closer to the surface of the plaque (instead of deeper and further away from the lumen). Supporting this, we found most of the Por-HDL-NP fluorescent signals were within the focal length of probe detection.

In this study, we utilized a GRIN fiber lens design for the miniaturized optics on the fiber-optic intravascular probe, similar to that previously reported by Scolaro et al.^33^. In this design, the same lens is used for both collection of fluorescence and OCT data, which is not optimal as these two imaging modalities have different optical requirements in terms of numerical aperture. The fluorescence signal could potentially be acquired with greater sensitivity using a higher numerical aperture lens that has been optimized for fluorescence capture alone. However, the OCT acquisition requires the use of a low numerical aperture lens to maximize imaging range. An improved lens design for this type of fiber-optic intravascular probe could involve a 3D printed lens-in-a-lens system^34^, where there is an outer lens with a high numerical aperture optimized for the collection of the fluorescence emission light beyond 800 nm, and a low numerical aperture inner lens optimized for OCT imaging to correct non-chromatic aberration. However, there are challenges in the utilization of such 3D printed lens systems for imaging the Por-HDL-NPs. The 3D printing photoresist material (IP-S) that has been previously used for fabrication of such a lens^34^ has been shown to suffer from autofluorescence when using the laser excitation wavelength for the nanoparticle (660 nm)^35^. Such autofluorescence would confound accurate measurement of fluorescence from the nanoparticle. Further optimization regarding the photoresist material is required if such a lens-in-a-lens system is applied for nanoparticle fluorescence detection. With that, it holds promise for more effective and sensitive collection of fluorescence indicative of disease progression.

In conclusion, this paper has explored Por-HDL-NPs for their fluorescence capacity and ability to assist with the detection of atherosclerotic plaques. We found that systemically infused Por-HDL-NPs track to murine atherosclerotic plaque and are detectable using our dual-modality intravascular imaging probe. Notably, by virtue of their highly fluorescent properties, Por-HDL-NPs exhibited application for the detection of early-stage plaque. Measurements captured by the intravascular probe for both plaque fluorescence and size were strongly correlated with those captured by traditional histological analyses on co-registered sections. Our findings have significant implications for the use of fluorescent, plaque-targeting Por-HDL-NPs for the accurate detection of early-stage atherosclerotic disease using dual-modality intravascular imaging approaches.

## Methods

### System setup

**Figure 6:**
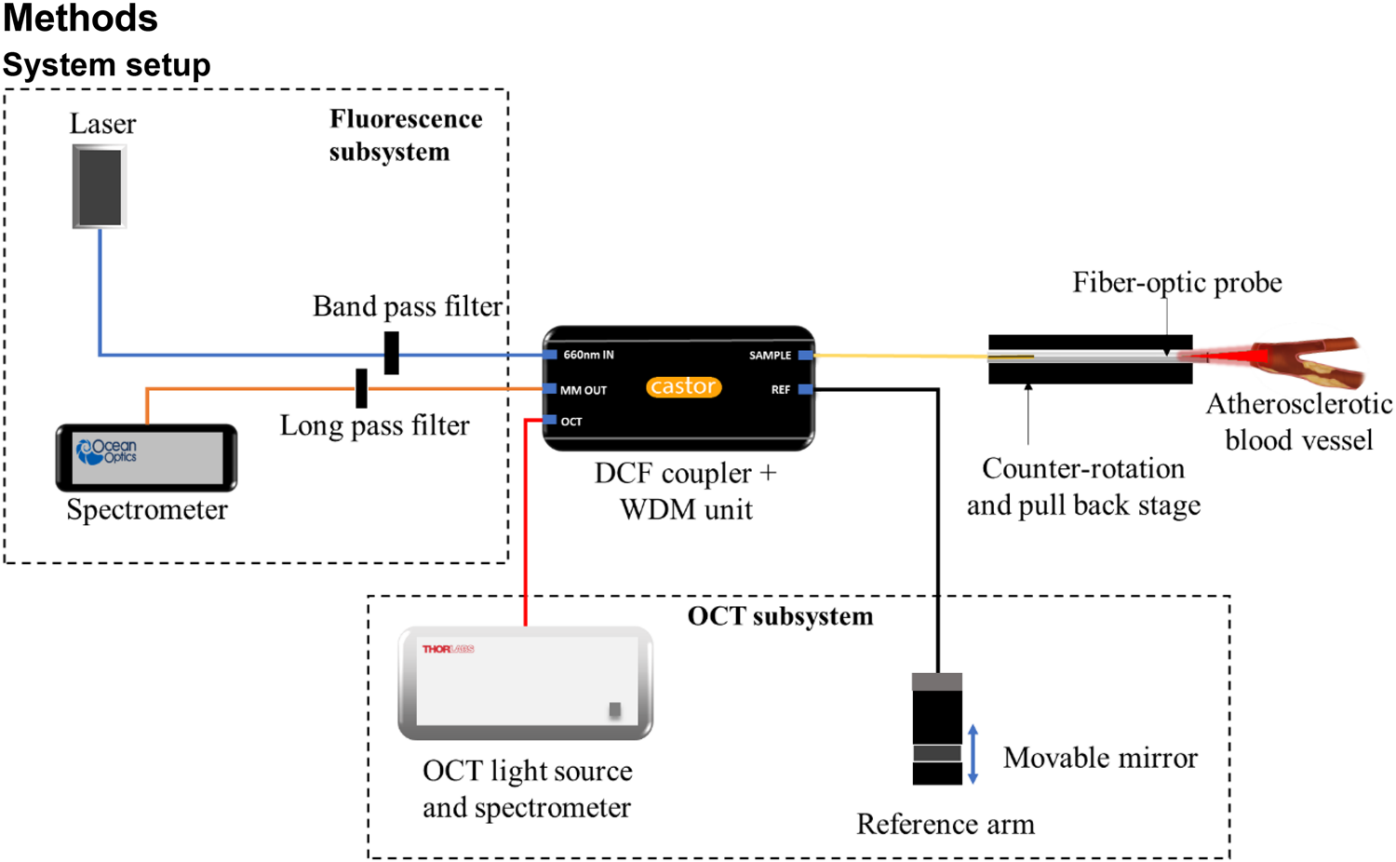
OCT + fluorescence system schematic; The OCT and fluorescence sub-systems are combined using a double-clad fiber (DCF) coupler module which contains DCF coupler and wavelength division multiplexer (WDM) unit.

The system setup is illustrated in Fig. 6. This setup contains two subsystems. The first is a spectral-domain OCT scanner, comprising of a light source, spectrometer, and data acquisition card (1300 nm central wavelength, Telesto II, ThorLabs GmbH, Germany), and a custom-built reference arm which consists of a movable mirror and a FiberPort collimator (PAF2-A4C, Thorlabs Inc., USA). The second subsystem is a fluorescence imaging unit. This consists of a 660 nm excitation laser (Matchbox, Integrated Optics, UAB) with a 656 nm bandpass filter (Edmund Optics Inc., USA) to further narrow the spectrum of the excitation light; and a 664 nm long pass filter (Semrock Inc., USA) to filter the detected fluorescence emission light, coupled to a spectrometer (QEPro, Ocean Insight, USA) to record the spectrum of the fluorescence emission light. The OCT and fluorescence subsystems are combined using a double clad fiber coupler module (DC1300LE2, Castor Optics Inc., Canada), with the OCT and fluorescence light beams then coupled into the dual-modality intravascular probe.

The fiber-optic intravascular probe comprises of a length of double-clad fiber (DCF13, ThorLabs Inc., USA), proximally connected to the double clad fiber coupler module and with focusing-optics fabricated onto the distal end. The focusing optics are fabricated by splicing precise lengths of different types of optical fiber onto the DCF. Specifically, the DCF was distally terminated with a 350 μm length of no-core fiber (NCF125, Success Prime Corporation, Taiwan) to allow the light beam to expand, and 150 μm length of GRIN fiber (DrakaElite 100/125 μm, Draka Communications Inc., USA) to weakly focus the beam, and then a length of no-core fiber angle-polished at 52° to redirect the light beam sideways using total internal reflection at the fiber-air interface. The optical fiber was then fitted into a metal torque coil (ACTONE, ASAHI Intecc Co., Ltd., Japan) for reliable rotation of the probe, and then encased within a transparent plastic catheter sheath (material: fluorinated ethylene propylene, 400 μm internal diameter, 635 μm outer diameter; ZEUS Inc., USA) to protect the vessel walls from trauma as the fiber-optic probe was rotated and pulled-back for imaging. The probe was rotated and translated with a custom-built counter-rotation and pullback stage at a rate of one rotation per second. During each rotation, 1600 A scans were acquired to form a radial OCT image, and 32 A scans were acquired for the fluorescence for each rotation. The detected OCT and fluorescence signals were recorded by a computer workstation, and the combined OCT + fluorescence image was reconstructed using custom software implemented in the C++ language.

### In vitro study: confocal fluorescence measurement

Cultured iBMDMs were plated onto coverslips in a 6-well plate with 3 × 10^6^ cells per well. The iBMDMs were incubated with 10 µM of Por-HDL-NPs per well or an equal volume of PBS vehicle control for 24 h. Por-HDL-NPs were synthesized in Toronto, Canada, according to previously published method^11^. Details of the nanoparticle such as the formulation, size, and the ligand density were described previously^15^. The cells were mounted onto glass slides using VECTASHIELD® Antifade Mounting Medium with DAPI (Vector Laboratories, US) for imaging under the confocal system (Leica TCS SP8X/MP, Leica, Germany). Images were acquired with 20x magnification, 660 nm for laser excitation and 700 nm and above for fluorescence emission collection. Cells imaged using the intravascular probe used Aquatex® mounting medium (Sigma-Aldrich, US) without DAPI stain to avoid fluorescence from DAPI being detected by the probe.

### Intravascular imaging study: murine atherosclerosis model

All experiments and procedures were approved by the South Australian Health and Medical Research Institute (SAHMRI) Animal Ethics Committee (AEC application SAM422.19) and conformed to the Australian code for the care and use of animals for scientific purposes and abided by the ARRIVE guidelines. Male Apoe^-/-^ mice were fed a high cholesterol diet containing 21% fat and 0.15% cholesterol (SF-00219, Semi-Pure Rodent Diet, Specialty Feeds) for 20 weeks. This induces atherosclerotic plaque growth in the descending aorta. For the experimental group (n = 2), the mice were intravenously injected with 48mg/kg of Por-HDL-NPs through the tail vein. For the negative control group (n = 1), an equal volume of PBS was administered through the tail vein. Eighteen hours after injection the mice were humanely killed by cardiac puncture and the vasculature was flushed with saline to remove residual blood in the descending aorta.

The thoracic aorta was imaged using the intravascular probe. During imaging, the probe was rotated and pulled back at 20 µm steps by the counter-rotation and pullback translation stage to image the vessel region which contained plaque.

The aorta was excised after intravascular imaging and embedded in optimal cutting temperature medium for fresh frozen cryosectioning. The aorta was sectioned at 5 μm sections consecutively for 8 slides, with 4 sections for each slide. This was done so that the OCT images could be matched to each of the sections within the same slide. Then 60 μm of tissue was discarded, which is equivalent of 3 OCT images. This process was repeated until the entire aorta was sectioned.

Histology was performed post imaging, stains included H & E and DAPI for cell nuclei. Immunofluorescence staining was performed to detect the number of CD68^+^ (MCA1957GA Bio-Rad) macrophages. Stained sections were scanned using a slide scanner (ZEISS Axio Scan.Z1 Slide Scanner, Carl Zeiss AG, Germany), with 385 nm, 475 nm and 630 nm light source for imaging the DAPI (Fig. 3a, c–e, g, h), CD68 (Fig. 3b–d, f–h) and Por-HDL-NPs (Fig. 3a, c, d) fluorescence respectively.

### Data analysis

To quantify the comparison between the intravascular imaging and the gold standard histology, we have manually co-registered the intravascular fluorescence with corresponding fluorescence microscopy images, as well as intravascular OCT with corresponding H & E images. The co-registration of the histology images and intravascular images was through matching of the pull-back distance in the intravascular image sequence with the position of histology sections; and the identification of anatomical landmarks, for example, matching the location of bifurcations and the number and locations of plaques within the artery. A MATLAB (MathWorks, USA) program was written to calculate the percentage of the circumference of the lumen that showed 720 nm fluorescence emission above the threshold value of 30 intensity units per rotation. The threshold was empirically selected after inspecting several images. The same threshold value was used across all data sets to ensure comparable quantification of the degree and extent of fluorescence. To quantify the extent of fluorescence around the circumference of the lumen for the immunofluorescence-stained sections, the following process was followed using the image analysis software, Fiji^36^:

1. Each fluorescence microscopy image was displayed, and the lumen was manually delineated to provide an estimate of total lumen circumference.
2. The violet channel of the fluorescence microscopy images was thresholded to identify areas with a positive fluorescence signal. The same threshold value was used across all images.
3. Locations on the lumen with a positive fluorescence signal were manually delineated, and the total length of these regions was summed.
4. The radial fluorescence measurement was computed as the ratio of the length of positive fluorescence regions (step 3) to the total internal circumference of the lumen (Step 1).

We have plotted the fluorescence measurements from intravascular images and fluorescence microscopy along the length of the vessel to visually illustrate the change in fluorescence signals along the length of the aorta as well as showing the similarities between the quantification from the two measurements (Fig. 4).

Similarly, for intravascular OCT and its manually co-registered corresponding H & E sections, plaque size was measured by the percentage of the circumference of the vessel lumen. Analysis of the OCT images was completed using custom software implemented in MATLAB (MathWorks, USA). For each intravascular OCT image, the number of A scans which correspond to the presence of a plaque (visualized by intimal thickening) was divided by the total number of A scans per rotation. The plaque size for the corresponding H & E sections was reported in the form of plaque area. Measurements on H & E sections were obtained by tracing around the plaque in NDP.View 2 software (Hamamatsu, Japan). H & E sections that were torn during histological processing were excluded from the quantification analysis. Statistical analyses were computed using Original Lab software (OriginLab, US). A *p* value < 0.001 was considered statistically significant for linear correlation analysis between two measurement groups.

## Data Availability

The datasets generated during and/or analyzed during the current study are available from the corresponding author on reasonable request.
